# Subperiosteal Implant Application in Full‐Jaw Reconstruction for Severely Atrophic Ridge: A Case Report

**DOI:** 10.1155/crid/3710212

**Published:** 2026-07-26

**Authors:** Mohammad Amin Bafandeh, Reza Amirzargar, Maryam Jahangiri, Sayed Shojaedin Shayegh

**Affiliations:** ^1^ Department of Prosthodontics, Faculty of Dentistry, Shahed University, Tehran, Iran, shahed.ac.ir; ^2^ Department of Oral and Maxillofacial Surgery, Faculty of Dentistry, Tehran University, Tehran, Iran, ut.ac.ir

**Keywords:** computer-aided design, dental implants, dental prosthesis implant-supported, jaw edentulous, prosthodontics

## Abstract

**Background:**

In cases of severe jaw atrophy, conventional implant‐supported prosthetic rehabilitation is often limited by insufficient bone volume and the need for extensive grafting procedures. These challenges can restrict prosthetic design and compromise functional outcomes. Customized subperiosteal implants, designed to fit directly over the existing bone surface, offer an alternative approach that enables full‐arch prosthetic reconstruction without bone augmentation.

**Methods:**

A patient with severe maxillary and mandibular atrophy underwent full‐arch rehabilitation using a customized subperiosteal implant fabricated through CAD/CAM technology based on three‐dimensional imaging. Although the implant framework was digitally designed, the prosthetic phase utilized conventional impression techniques to accurately capture soft tissue and abutment details. The definitive prosthesis was fabricated using a traditional laboratory workflow focusing on optimal occlusion, esthetics, and patient comfort. A structured clinical timeline, detailed surgical protocol, and short‐term clinical and radiographic follow‐up were documented to ensure reproducibility and objective outcome assessment.

**Results:**

The customized subperiosteal framework provided stable support for the final prosthesis. Conventional impression methods allowed precise adaptation of the restoration, minimal chairside adjustment, and satisfactory esthetic and functional outcomes. At 6‐month follow‐up, no implant mobility, soft‐tissue exposure, infection, or prosthetic complications were observed. Clinical examination demonstrated healthy peri‐implant soft tissue and stable radiographic findings. The patient reported high satisfaction with mastication, speech, and comfort.

**Conclusion:**

Customized subperiosteal implants, even when combined with conventional prosthetic techniques, can reliably support full‐arch restorations in patients with severely resorbed jaws. This approach merges digital surgical design with traditional prosthodontic principles, providing predictable, efficient, and patient‐centered rehabilitation when conventional implant placement is not feasible. Interdisciplinary planning and strict quality‐control protocols are essential to prevent design errors and optimize outcomes.

**Summary**

• Interdisciplinary surgeon–prosthodontist collaboration is essential
• Prosthetic‐driven implant design prevents major complications
• Overdenture conversion can successfully manage non‐parallel abutments
• Structured follow‐up is critical for soft‐tissue monitoring

## 1. Introduction

Today, dental implants are considered the primary option for replacing missing teeth [1]. Various types of dental implants have been introduced, including endosseous, transosseous, and subperiosteal implants [2]. Endosseous implants are the most used; however, anatomical limitations, severe bone resorption, or pathological conditions that lead to extensive bone loss may restrict their use [3]. In such cases, autogenous bone grafts harvested from other parts of the body or guided bone regeneration procedures are among the clinician’s available approaches for reconstructing the deficient area prior to implant surgery. Nevertheless, these methods present challenges such as prolonged treatment duration, tissue rejection, and a risk of treatment failure [4].

Subperiosteal implants represent an alternative that may be indicated in these complex situations. Although these implants were first introduced in the 1940s, they received relatively little attention until approximately 15 years ago [2, 5]. Recent advances in imaging techniques and digital dentistry have facilitated more accurate and predictable workflows for the fabrication and placement of subperiosteal implants, making them a viable treatment option for patients with severely atrophic ridges [3, 5].

Subperiosteal implants are custom‐made using CAD‐CAM technology. Initially, a three‐dimensional intraoral scan of the patient’s dentition—prior to extraction, or of the temporary denture—is obtained. Subsequently, the patient’s CBCT data and intraoral records are integrated within specialized design software. This allows for the fabrication of a customized subperiosteal implant tailored to the planned final prosthesis, incorporating appropriate supporting arms for optimal prosthetic stability [6].

These implants typically feature a lattice or mesh structure, which reduces overall weight while promoting bone growth between the implant components [3]. The mesh framework is placed beneath the gingiva and onto the surface of the jawbone. For primary stability, the implant is secured to the bone with screws, after which the gingival tissues are repositioned to cover the site [5].

Recent biomechanical investigations using finite element analysis have provided further insight into the mechanical behavior of subperiosteal implants in severely atrophic jaws. In a study based on realistic three‐dimensional models reconstructed from DICOM data and converted into STL files, different implant configurations and loading scenarios were analyzed to evaluate stress distribution within the implant–bone complex. The findings demonstrated that load application in anterior regions generates the highest stress concentrations, particularly around cortical fixation screws, whereas optimized designs—such as the inclusion of posterior fixation and improved structural connections—can significantly enhance load distribution and reduce critical stress peaks. Moreover, the metallic framework of subperiosteal implants was shown to withstand masticatory forces without structural failure, and no significant differences were observed between one‐piece and two‐piece designs. Importantly, the prosthetic superstructure plays a crucial role in reinforcing the overall rigidity of the system. These findings highlight the importance of prosthetic‐driven design and proper fixation strategies in improving the biomechanical performance and long‐term success of subperiosteal implants, although further clinical studies are required to validate these results [7].

More recently, a systematic review of finite element studies on subperiosteal implants has further expanded the understanding of their biomechanical behavior. This review demonstrated that subperiosteal implants tend to distribute occlusal forces more homogeneously compared with conventional endosseous implants, resulting in reduced peak stress at the bone–implant interface. However, their mechanical performance is highly dependent on design‐related factors such as implant configuration, thickness, screw fixation, and material properties. In particular, dual or multiunit configurations were associated with improved stress distribution, while reduced implant thickness and oblique loading conditions significantly increased stress concentration and displacement risk. In addition, material selection was shown to influence biomechanical outcomes, with titanium providing superior structural stability, whereas alternative materials such as PEEK may reduce implant stress but potentially increase stress transfer to the surrounding bone. Overall, this evidence supports the importance of design optimization and load management in enhancing the biomechanical reliability of subperiosteal implants [8].

Because the success of full‐arch rehabilitation using subperiosteal implants depends equally on both the surgical and prosthetic phases—and considering the limited literature that describes each step of the prosthetic workflow in detail—this case report presents a comprehensive overview of the prosthetic procedures involved in fabricating an optimal final prosthesis for a patient who received a subperiosteal implant due to severe ridge atrophy.

## 2. Clinical Report

The patient is a 60‐year‐old man who underwent placement of subperiosteal implants due to a severely atrophic ridge and the impossibility of performing autogenous bone grafting procedures. Previously, the patient had a mandibular overdenture supported by two implants and a complete maxillary denture. Over time, progressive maxillary resorption and increased mobility of the mandibular denture resulted in patient discomfort. The patient was comprehensively examined by an oral and maxillofacial surgeon.

Preoperative assessment included panoramic radiography (Figure 1), intraoral examination (Figure 2), extraoral facial evaluation (Figure 3), and fabrication of a plastic prototype of the designed subperiosteal prosthesis (Figure 4). After evaluating the case, the surgeon designed customized subperiosteal implants for the patient, which were placed in a single surgical session in the operating room.

**Figure 1:**
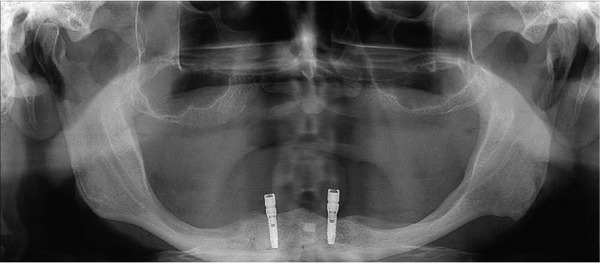
Preoperative panoramic radiograph demonstrating severe maxillary atrophy and existing mandibular implants.

**Figure 2:**
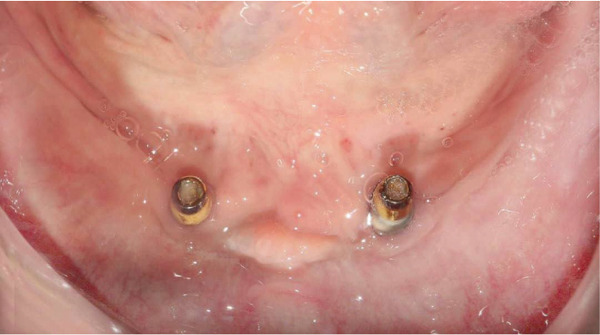
Preoperative intraoral view showing edentulous maxilla and mandibular overdenture condition.

**Figure 3:**
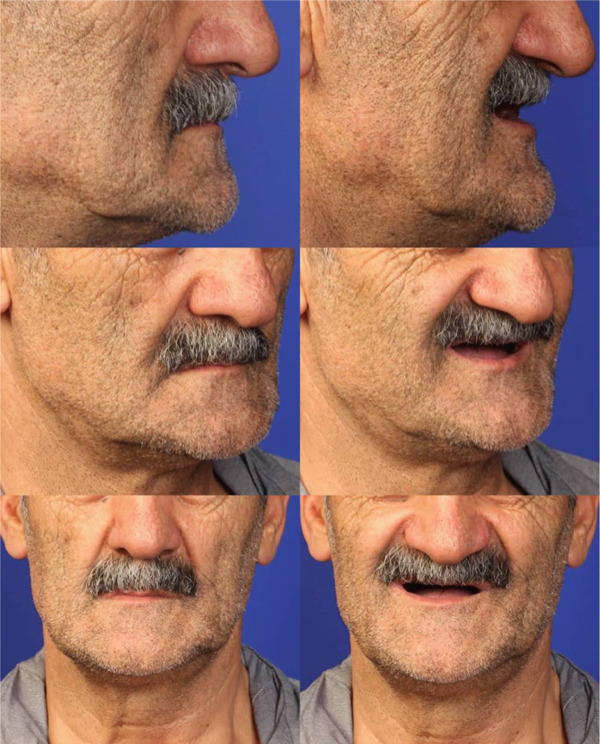
Preoperative extraoral views illustrating facial profile and vertical dimension.

**Figure 4:**
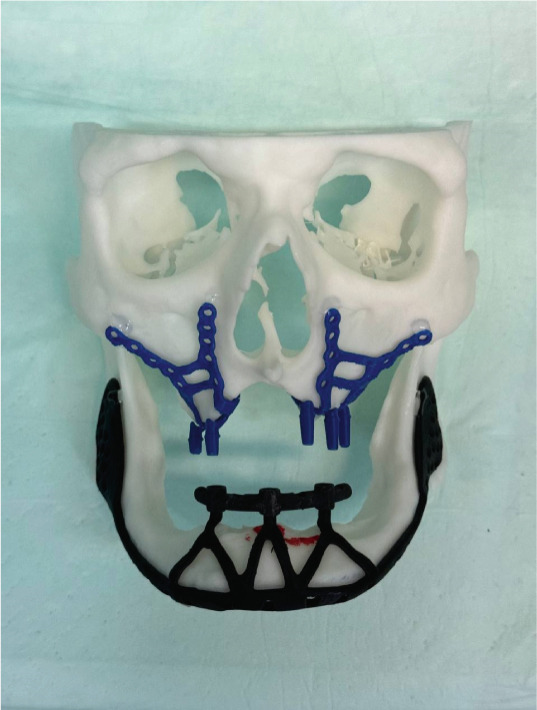
Plastic prototype of the patient‐specific subperiosteal implant design used for presurgical evaluation.

In the present case, prosthetic treatment was intentionally postponed for 6 months to allow complete soft‐tissue healing and biological stabilization following extensive subperiosteal implant surgery. This healing period likely contributed to the absence of early biological complications observed during follow‐up.

Figure 5 shows the postoperative panoramic radiograph. Based on the surgeon’s clinical judgment, the existing endosseous implants were not removed and were instead left submerged beneath the gingiva. In the mandible, the subperiosteal implant derives support from both rami and the inferior border of the mandible, with retention achieved through screws in the ramus regions. In the maxilla, support and retention are obtained from the zygomatic arch and the piriform region.

**Figure 5:**
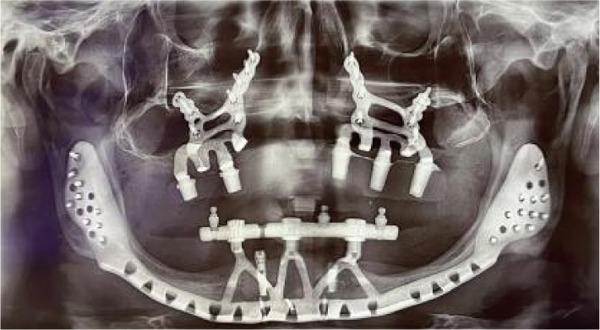
Postoperative panoramic radiograph showing placement of customized subperiosteal implants in the maxilla and mandible.

In Figure 6, the intraoral view of the maxillary and mandibular subperiosteal implant‐supported prostheses is shown. In the maxilla, five abutments were placed for prosthetic support in the regions corresponding to Teeth 4 and 6 on both sides, as well as the left canine. In the mandible, the framework was designed in the form of a bar‐and‐wing structure.

**Figure 6:**
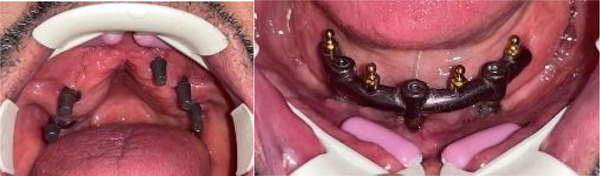
Intraoral view of the subperiosteal implant‐supported prostheses.

## 3. Clinical Timeline

A structured timeline of treatment is presented in Table 1.

**Table 1:** Timeline of treatment.

Stage	Procedure	Time
T0	Subperiosteal implant surgery	Day 0
T1	Initial impression	24 weeks postsurgery
T2	Final impression	25 weeks
T3	VD/CR registration	26 weeks
T4	PMMA try‐in	27weeks
T5	Prosthesis delivery	28 weeks
Follow‐up	Clinical and radiographic exam	3 and 6 months

### 3.1. Digital Design and Manufacturing Workflow

CBCT imaging was obtained using a high‐resolution protocol (voxel size ≈0.2–0.3 mm) to accurately capture maxillofacial anatomy. DICOM data were merged with surface scan STL files using implant design software. The design was performed using commercially available dental implant CAD software according to the manufacturer’s protocol. The customized framework was digitally designed to maximize cortical bone support and distribute occlusal forces.

The implant was manufactured from medical‐grade titanium alloy (Ti‐6Al‐4V) using metal 3D printing/laser sintering. Postprocessing included polishing, heat treatment, and steam autoclave sterilization. Quality‐control procedures involved digital fit verification and physical model testing prior to surgery.

The previously noted design error (nonparallel maxillary abutments) was attributed to insufficient prosthetic consultation during the digital planning phase. Preventive quality‐control checkpoints should include a prosthodontic review of abutment angulation and prosthetic space before final manufacturing.

### 3.2. Surgical Protocol

Under general anesthesia, full‐thickness mucoperiosteal flaps were elevated to expose the alveolar bone. The customized framework was passively adapted and verified intraoperatively. Fixation screws were placed in predetermined cortical regions to achieve primary stability. Careful periosteal management was performed to minimize soft‐tissue tension.

Flaps were repositioned and sutured using interrupted sutures. Primary closure without tension was achieved. Sutures were removed after 10–14 days. Postoperative management included antibiotics, analgesics, and oral hygiene instructions. Early healing was uneventful, with no signs of infection or wound dehiscence.

### 3.3. Mandibular Design Rationale

The extended mandibular design utilizing bilateral ramus and inferior border support was selected to distribute occlusal loads across a larger cortical surface area and enhance biomechanical stability in severely atrophic bone. This extensive anchorage reduced stress concentration and improved resistance to functional forces. Screw placement was planned to avoid vital anatomical structures while maximizing fixation in high‐density cortical regions.

### 3.4. Prosthetic Procedures (Expanded Clarity)

1. Fabrication of an initial alginate impression (Kulzer, Germany) for the purpose of constructing a custom tray.
2. Fabrication of a custom tray with spacer.

Addition silicone impression materials (light‐body and heavy‐body) (Panasil, Kettenbach, Germany) and tray adhesive (Panasil, Kettenbach, Germany) were used to ensure dimensional accuracy. The impression was taken at the level of the subperiosteal abutments using a transfer technique (Figure 7).

3. After obtaining the master cast, a record base and wax rim were fabricated to register the maxillomandibular relationship. To achieve the necessary retention, the existing abutments in the mouth were engaged (Figure 8).
4. Using the record base and wax rim and after adjustment of the vertical dimension (VD), the maxillomandibular relationship was recorded in centric relation (CR) (Figure 9). In Figure 10, the VD before and after adjustment is shown.
5. Based on the adjusted VD, an initial PMMA trial prosthesis was fabricated and evaluated intraorally. Due to the lack of parallelism among the abutments and the patient’s excessive crown height space (CHS), an implant‐supported overdenture treatment plan was selected instead of a fixed prosthesis (Figure 11).
6. To achieve proper parallelism of the abutments and provide suitable attachment for the overdenture, metal copings were designed on the abutments, with wings incorporated into them. These copings were trialed on the maxillary abutments to ensure proper seating and fit. Metal copings were fabricated to correct abutment angulation and provide overdenture attachment support (Figure 12).
7. Finally, maxillary and mandibular overdentures were designed and fabricated for the patient. At the delivery appointment, the metal copings were first cemented onto the abutments using a permanent resin cement. The maxillary and mandibular overdentures were then seated intraorally, adjusted as necessary, and delivered to the patient (Figure 13).

**Figure 7:**
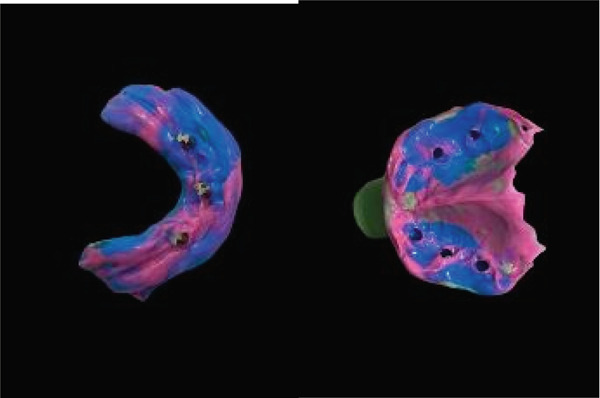
Final impression of both arches.

**Figure 8:**
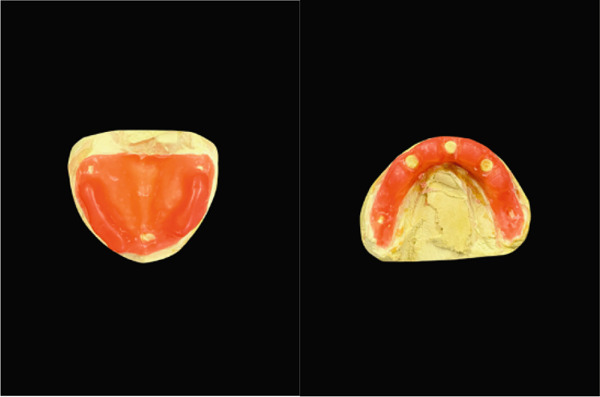
Maxillary and mandibular record base and wax rim.

**Figure 9:**
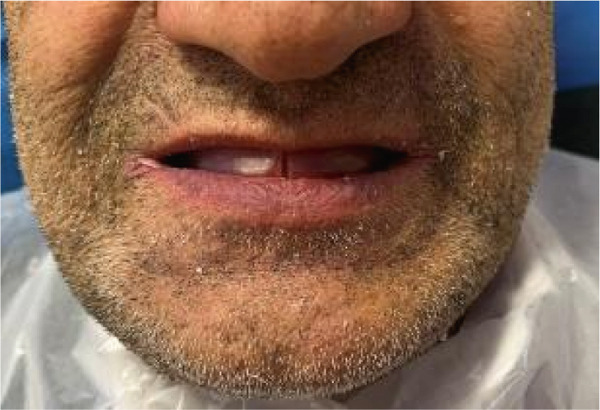
Maxillomandibular relationship record.

**Figure 10:**
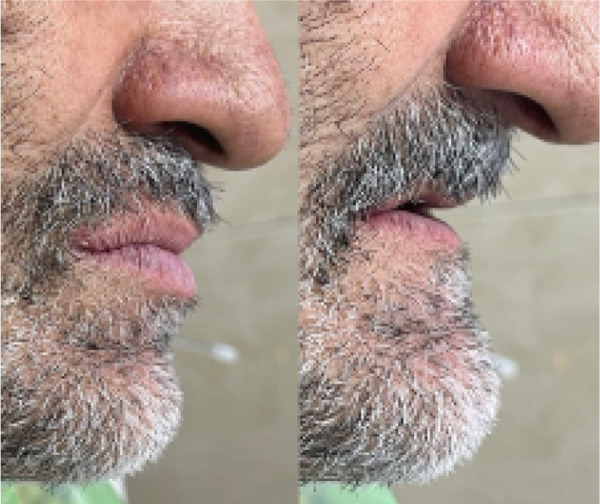
Adjustment of the patient’s vertical dimension.

**Figure 11:**
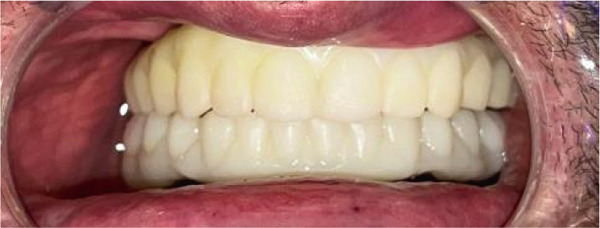
Intraoral try‐in of the PMMA denture.

**Figure 12:**
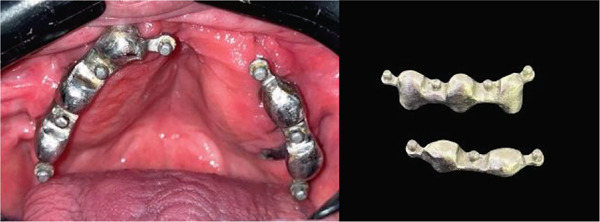
Metal copings designed for the maxillary overdenture.

**Figure 13:**
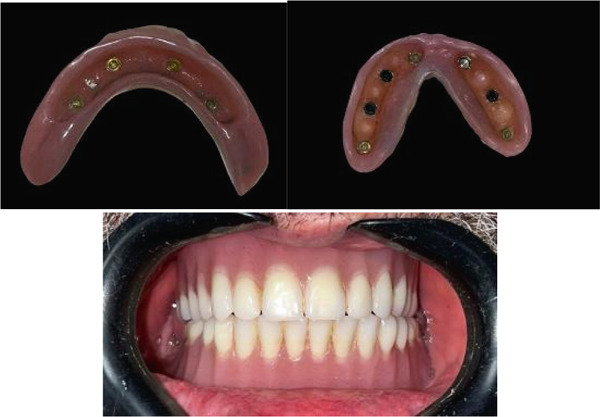
Final intraoral view of the patient’s overdentures.

### 3.5. Follow‐Up and Clinical Outcomes

At 3‐ and 6‐month follow‐up visits, clinical examination showed healthy keratinized tissue, absence of inflammation, and no soft‐tissue exposure. Radiographic evaluation demonstrated stable implant positioning. The patient reported improved mastication, speech, and overall satisfaction. No early or late biological or mechanical complications were observed during the observation period.

## 4. Discussion

In recent years, customized subperiosteal implants have emerged as a viable alternative for the rehabilitation of severely atrophic ridges, particularly when conventional endosseous implant placement is not feasible [9–12]. These implants eliminate the need for extensive bone grafting procedures, such as iliac crest augmentation, thus reducing treatment morbidity and overall duration [12]. Successful outcomes, however, depend not only on precise implant design, fabrication, and surgical placement but also on meticulous prosthetic planning and execution [13].

Recent clinical evidence further supports the growing role of subperiosteal implants in such complex scenarios. A systematic review and meta‐analysis reported an overall survival rate of approximately 91% for subperiosteal implants used in full‐arch rehabilitation of severely atrophic jaws, with significantly higher survival observed in implants placed using a single‐stage approach (100%) compared to two‐stage procedures (85%). In addition, complication rates were lower in single‐stage protocols (8.7%) compared to two‐stage approaches (20.8%), with fewer severe complications such as paresthesia and implant exposure. These findings have been largely attributed to advances in digital workflows, including CAD‐CAM fabrication and improved imaging techniques, which enhance implant fit and reduce surgical complexity [14].

In the present case, the patient had previously worn a mandibular overdenture supported by two implants and a complete maxillary denture. Progressive resorption of the maxilla and increased mobility of the mandibular denture led to fracture of the existing mandibular fixtures, necessitating full‐arch reconstruction with customized subperiosteal implants. The implants were placed in a single surgical session, and prosthetic rehabilitation was intentionally delayed for 6 months to allow complete soft‐tissue healing and stabilization of the framework.

This treatment approach is consistent with current trends favoring single‐stage, digitally planned subperiosteal implants, which have been associated with improved clinical outcomes and reduced complication rates.

A key challenge in this case was the initial design of nonparallel maxillary abutments, which, combined with excessive CHS, required modification of the treatment plan from a fixed prosthesis to an overdenture. The fabrication of metal copings enabled correction of abutment angulation and provided reliable retention for the overdenture. This experience underscores the critical importance of prosthetic‐driven planning and early interdisciplinary collaboration between surgeons and prosthodontists to prevent design‐related complications and ensure optimal functional outcomes.

Furthermore, previous biomechanical analyses have emphasized the importance of optimal load distribution and framework design in subperiosteal implants, highlighting that improper stress concentration—particularly in anterior regions or around fixation screws—may compromise long‐term outcomes. Therefore, prosthetic design decisions, such as abutment alignment and occlusal scheme, play a crucial role in minimizing biomechanical complications and enhancing implant longevity.

At 6‐month follow‐up, clinical and radiographic assessments demonstrated stable implant positioning, healthy peri‐implant soft tissue, absence of inflammation or exposure, and satisfactory patient‐reported outcomes in terms of mastication, speech, and comfort. These findings are consistent with recent literature reporting favorable short‐term outcomes and low complication rates for modern additively manufactured subperiosteal implants [11–13].

Key educational points from this case include the following:

1. Early prosthodontic consultation is essential during digital implant design.
2. Nonparallel abutments can necessitate treatment plan modification.
3. Structured follow‐up is critical to monitor soft‐tissue health and prosthetic stability.
4. Careful consideration of occlusal load distribution and framework support is vital, particularly in severely atrophic mandibles.

Limitations of this report include its single‐case design and relatively short follow‐up, which preclude generalization of findings. In addition, the current body of evidence is limited by heterogeneity among studies and relatively short follow‐up periods in modern single‐stage approaches, which should be considered when interpreting the results. Future studies with larger cohorts and longer observation periods are needed to validate these outcomes and refine prosthetic protocols for customized subperiosteal implants.

## 5. Conclusion

Subperiosteal implants represent a valuable treatment option for patients with severely atrophic ridges who are not candidates for extensive bone grafting procedures. This case demonstrates that, in addition to precise surgical placement, careful planning and execution of the prosthetic phase are critical to achieving optimal outcomes. Customized design, combined with innovative solutions such as metal copings to correct abutment angulation, can allow for successful rehabilitation even when initial implant placement presents challenges.

Consistent with current clinical evidence, digitally designed subperiosteal implants placed in a single surgical phase appear to offer high survival rates and reduced complication profiles, making them a promising alternative for full‐arch rehabilitation in severely resorbed jaws.

The use of maxillary and mandibular overdentures supported by subperiosteal implants provided the patient with satisfactory esthetics, function, and speech, highlighting the importance of a multidisciplinary approach in complex implant cases. Although favorable outcomes were observed in this patient, the findings should be interpreted within the limitations of a single case report. Further well‐designed clinical studies, particularly randomized controlled trials with long‐term follow‐up, are required to confirm the predictability and generalizability of this treatment approach.

## Author Contributions

**Mohammad Amin Bafandeh:** prosthetic treatment, data curation. **Reza Amirzargar:** surgical procedures. **Maryam Jahangiri:** conceptualization, writing – original draft, writing – review and editing. **Sayed Shojaedin Shayegh:** supervision, writing – review and editing.

## Funding

No funding was received for this manuscript.

## Disclosure

After using this tool and service, the authors reviewed and edited the content as needed and took full responsibility for the content of the publication.

## Ethics Statement

This case report was conducted in accordance with the principles of the Declaration of Helsinki (latest revision, 2024). As this is a single‐case report of routine clinical care, formal institutional review board (IRB) approval was not required. Written informed consent was obtained from the patient for treatment and publication, including clinical images and relevant data.

## Conflicts of Interest

None of the authors have a conflict of interest to disclose

## Supporting information


**Supporting Information** Additional supporting information can be found online in the Supporting Information section. Completed CARE checklist used for reporting this case report according to the CARE guidelines.

## Data Availability

The data that support the findings of this study are available from the corresponding author upon reasonable request.
