# Supplementary material for: Subperiosteal Implant Application in Full‐Jaw Reconstruction for Severely Atrophic Ridge: A Case Report
Source: Case Rep Dent. 2026 Jul 26;2026:3710212. doi: 10.1155/crid/3710212 (PMC13402887; doi:10.1155/crid/3710212)
Supplement: Supplementary file 1 — Supporting Information Additional supporting information can be found online in the Supporting Information section. Completed CARE checklist used for reporting this case report according to the CARE guidelines. [file CRID-2026-3710212-s001.docx]

**CARE Case Report Checklist**

| **CARE Item** | **Description** | **Reported in Manuscript (Section/Page)** |
| --- | --- | --- |
| Title | The words “case report” are included in the title and describe the main focus of the case | Title page |
| Keywords | 2–5 key words identifying key elements of the case | Abstract |
| Abstract – Introduction | What is unique about this case and what it adds to the literature | Abstract – Background |
| Abstract – Case Presentation | Main symptoms, clinical findings, diagnosis, interventions, and outcomes | Abstract – Methods/Results |
| Abstract – Conclusion | Main take-away message | Abstract – Conclusion |
| Introduction | Brief background and rationale for reporting the case | Introduction |
| Patient Information | Demographic details and relevant medical history | Clinical Report |
| Clinical Findings | Relevant physical examination findings | Clinical Report |
| Timeline | Chronological table of events and interventions | Clinical Timeline Table |
| Diagnostic Assessment | Diagnostic methods and reasoning | Clinical Report / Digital Workflow |
| Therapeutic Intervention | Surgical and prosthetic procedures | Surgical Protocol / Prosthetic Procedures |
| Follow-Up and Outcomes | Clinical and radiographic outcomes, complications | Follow-Up and Clinical Outcomes |
| Discussion – Strengths & Limitations | Interpretation of findings and limitations | Discussion |
| Discussion – Literature Context | Comparison with relevant literature | Discussion |
| Discussion – Take-Home Messages | Key lessons from the case | Conclusion / Key Messages |
| Patient Perspective | Patient-reported satisfaction and experience | Follow-Up and Clinical Outcomes |
| Informed Consent | Confirmation of written patient consent | Declaration of Patient Consent |
| Ethical Approval | Statement of ethical compliance | Ethics Statement section |
